# Reduced Gray Matter Volume in the Social Brain Network in Adults with Autism Spectrum Disorder

**DOI:** 10.3389/fnhum.2017.00395

**Published:** 2017-08-04

**Authors:** Wataru Sato, Takanori Kochiyama, Shota Uono, Sayaka Yoshimura, Yasutaka Kubota, Reiko Sawada, Morimitsu Sakihama, Motomi Toichi

**Affiliations:** ^1^Department of Neurodevelopmental Psychiatry, Habilitation and Rehabilitation, Graduate School of Medicine, Kyoto University Kyoto, Japan; ^2^Brain Activity Imaging Center, Advanced Telecommunications Research Institute International Kyoto, Japan; ^3^Health and Medical Services Center, Shiga University Shiga, Japan; ^4^Rakuwa-kai Otowa Hospital Kyoto, Japan; ^5^Faculty of Human Health Science, Kyoto University Kyoto, Japan; ^6^The Organization for Promoting Neurodevelopmental Disorder Research Kyoto, Japan

**Keywords:** amygdala, autism spectrum disorder (ASD), fusiform gyrus, dorsomedial prefrontal cortex, middle temporal gyrus, social brain network

## Abstract

Autism spectrum disorder (ASD) is a neurodevelopmental disorder characterized by behavioral impairment in social interactions. Although theoretical and empirical evidence suggests that impairment in the social brain network could be the neural underpinnings of ASD, previous structural magnetic resonance imaging (MRI) studies in adults with ASD have not provided clear support for this, possibly due to confounding factors, such as language impairments. To further explore this issue, we acquired structural MRI data and analyzed gray matter volume in adults with ASD (*n* = 36) who had no language impairments (diagnosed with Asperger’s disorder or pervasive developmental disorder not otherwise specified, with symptoms milder than those of Asperger’s disorder), had no comorbidity, and were not taking medications, and in age- and sex-matched typically developing (TD) controls (*n* = 36). Univariate voxel-based morphometry analyses revealed that regional gray matter volume was lower in the ASD than in the control group in several brain regions, including the right inferior occipital gyrus, left fusiform gyrus, right middle temporal gyrus, bilateral amygdala, right inferior frontal gyrus, right orbitofrontal cortex, and left dorsomedial prefrontal cortex. A multivariate approach using a partial least squares (PLS) method showed that these regions constituted a network that could be used to discriminate between the ASD and TD groups. A PLS discriminant analysis using information from these regions showed high accuracy, sensitivity, specificity, and precision (>80%) in discriminating between the groups. These results suggest that reduced gray matter volume in the social brain network represents the neural underpinnings of behavioral social malfunctioning in adults with ASD.

## Introduction

Autism spectrum disorder (ASD) is a behaviorally defined neurodevelopmental disorder characterized primarily by impairment in social interactions (American Psychiatric Association, 2013). For example, several behavioral studies have reported that individuals with ASD, compared with typically developing (TD) individuals, exhibit less attention to others’ faces (Trepagnier et al., 2002), reduced emotional reactions (Yirmiya et al., 1989), and facial mimicry (Yoshimura et al., 2015) to others’ facial expressions, and reduced ability to read the others’ mental states from their eyes (Baron-Cohen et al., 2001).

Regarding the underlying neural mechanism of such behavioral social impairments, some researchers have proposed that a network of specific brain regions that are involved in processing social signals, called the “social brain” network (Brothers et al., 1990; Adolphs, 2003; Blakemore, 2008), may be impaired in individuals with ASD (Emery and Perrett, 2000; Johnson et al., 2005; Bachevalier and Loveland, 2006; Frith, 2007; Pelphrey and Carter, 2008). Although details differ among researchers regarding which brain regions are included in the network, the regions typically are said to include the inferior occipital gyrus (IOG), posterior fusiform gyrus (FG), posterior middle temporal gyrus (MTG, including its adjacent regions such as the superior temporal sulcus and gyrus; cf. Allison et al., 2000), amygdala, inferior frontal gyrus (IFG), orbitofrontal cortex (OFC, including both medial and lateral areas; cf. Rudebeck and Murray, 2011), and dorsomedial prefrontal cortex (DMPFC). Ample functional neuroimaging and neuropsychological evidence in TD individuals suggests that these social brain regions are related to specific processing for social stimuli, such as the visual analysis of faces in the IOG, FG, and MTG (for a review, see Haxby et al., 2000), emotional processing in the amygdala and OFC (for reviews, see Calder et al., 2001; Rolls, 2004), motor resonance in the IFG (for a review, see Rizzolatti et al., 2001), and mindreading in the DMPFC (for a review, see Frith and Frith, 2003). Consistent with this proposal, several previous functional neuroimaging studies in individuals with ASD have reported that during the processing of social stimuli, individuals with ASD show reduced activity in regions within the social brain network, such as the IOG (e.g., Deeley et al., 2007; Scherf et al., 2010; Sato et al., 2012), FG (e.g., Critchley et al., 2000; Schultz et al., 2000; Pierce et al., 2001), MTG (e.g., Critchley et al., 2000; Pelphrey et al., 2005; Williams et al., 2006), amygdala (e.g., Baron-Cohen et al., 1999; Critchley et al., 2000; Pierce et al., 2001), IFG (e.g., Baron-Cohen et al., 1999; Hall et al., 2003; Dapretto et al., 2006), OFC (e.g., Ashwin et al., 2007; Kana et al., 2009; Kaiser et al., 2010), and DMPFC (e.g., Castelli et al., 2002; Pierce et al., 2004; Wang et al., 2007) (for reviews, see Philip et al., 2012; Nickl-Jockschat et al., 2015). These theoretical and empirical findings suggest that the decreased activity in the social brain network underlies the behavioral social impairment in individuals with ASD.

To complement this understanding of the neural underpinnings of ASD, several structural magnetic resonance imaging (MRI) studies have investigated structural neural abnormalities in individuals with ASD. However, to date, the results have been inconsistent across studies (e.g., Abell et al., 1999; for reviews, see Duerden et al., 2012; Nickl-Jockschat et al., 2012; Yang et al., 2016). These inconsistent results may be at least partly explained by abnormal trajectories of brain development in ASD (for a review, see Sacco et al., 2015). However, even studies testing only adult participants have reported inconsistent findings (Abell et al., 1999; McAlonan et al., 2002; Hadjikhani et al., 2006; Schmitz et al., 2006, 2008; Craig et al., 2007; Wilson et al., 2009; Toal et al., 2010; Dziobek et al., 2010; Scheel et al., 2011; Ecker et al., 2012, 2013; Lai et al., 2013; Mueller et al., 2013; Bernhardt et al., 2014; Riedel et al., 2014; Balardin et al., 2015; Gebauer et al., 2015; Itahashi et al., 2015; Lai et al., 2015; Libero et al., 2015; for a review, see Yang et al., 2016). Although several studies reported reduced gray matter volume or cortical thickness in the social brain regions among individuals with ASD, including the IOG (Hadjikhani et al., 2006; Ecker et al., 2012), FG (Hadjikhani et al., 2006; Craig et al., 2007; Toal et al., 2010; Ecker et al., 2012), MTG (Hadjikhani et al., 2006; Craig et al., 2007; Scheel et al., 2011; Ecker et al., 2012; Mueller et al., 2013), amygdala (Craig et al., 2007; Lai et al., 2015), IFG (Hadjikhani et al., 2006; Mueller et al., 2013), OFC (Hadjikhani et al., 2006; Craig et al., 2007), and DMPFC (Abell et al., 1999; Hadjikhani et al., 2006), only a few studies reported problems in multiple social brain regions. Furthermore, no reported study has provided evidence as to whether the brain regions that exhibit reduced gray matter volume actually constitute the network in adults with ASD.

These inconsistent findings across structural MRI studies in adults with ASD may be explained by confounding factors such as language impairments, which are major and heterogeneous in ASD (for reviews, see Boucher, 2012; Mody and Belliveau, 2013). Language anomalies (e.g., language delay) are included in the diagnostic criteria of the International Classification of Diseases 10 (World Health Organization, 1992) and in the Diagnostic and Statistical Manual of Mental Disorders, Fourth Edition, Text Revision (DSM-IV-TR) (American Psychiatric Association, 2000); however, they are not included in the DSM-5 (American Psychiatric Association, 2013). For the first two diagnostic systems, individuals with and without language/communication impairments, in addition to the core ASD symptoms of social deficits and repetitive/restricted tendencies, are diagnosed with autistic disorder and Asperger syndrome/disorder, respectively. Some previous studies of individuals with ASD have revealed widespread changes in gray matter structures associated with language impairments (Lai et al., 2015; Sharda et al., 2017). For example, Sharda et al. (2017) found that poorer language ability was associated with greater cortical thickness in the temporal and frontal cortices. Some studies also compared the brain structure in children with autistic disorders and Asperger disorder/syndrome and reported at least partially discrete structural abnormalities (Kwon et al., 2004; McAlonan et al., 2008). Regarding this issue, the majority of the abovementioned structural MRI studies investigating ASD in adults (excluding McAlonan et al., 2002) included (at least partially) individuals with autistic disorders or did not report the subtypes of ASD of the participants. Therefore, it is possible that the structural abnormalities related to language impairments could overshadow those in the social brain regions. In addition, factors, such as comorbidity and medication, may act as confounding factors and have not been controlled or reported in some previous structural MRI studies in adults with ASD.

To provide further evidence regarding structural neural abnormalities in ASD, we acquired structural MRI data from adults with ASD who had no language impairments (diagnosed with Asperger’s disorder or pervasive developmental disorder not otherwise specified [PDD-NOS], with symptoms milder than those of Asperger’s disorder), had no comorbidities, and were not taking medication. We also tested age- and sex-matched TD controls. First, we analyzed group differences in regional gray matter volume using univariate voxel-based morphometry (VBM). The univariate VBM was used to identify the brain regions showing differences in gray matter volume between groups. Next, to identify group differences at a network level, we applied a multivariate approach and conducted a partial least squares (PLS) analysis (McIntosh et al., 1996). PLS analysis was used to identify the brain network (i.e., the sets of correlated voxels) that could be used to discriminate between the ASD and TD groups. We further conducted PLS discriminant analysis (PLS-DA) (Barker and Rayens, 2003) using information derived from the PLS analysis to determine the accuracy, sensitivity, specificity, and precision of the brain network in discriminating between groups. We also explored the association between the regional/network-level gray matter volume reduction and the severity of social malfunctioning related to ASD. Although direct evidence from previous structural MRI studies in ASD groups has not been consistent, based on results suggestive of an impaired social brain network in adults with ASD and our controlling for possible confounding factors, we predicted that the univariate VBM would show decreased regional gray matter volume in the social brain regions in the ASD group. We also predicted that the multivariate PLS and PLS-DA would identify the social brain network and reveal a correlated reduction in gray matter volume in ASD, which would differentiate between the ASD and TD groups with certain degree of accuracy, sensitivity, specificity, and precision, and would be correlated with the severity of social malfunctioning.

## Materials and Methods

### Participants

The ASD group consisted of 36 adults with ASD (11 females, 25 males; mean ± *SD* [range] age = 27.0 ± 8.0 [18–53] years). The group consisted of 21 (7 females, 14 males) individuals with Asperger’s disorder and 15 (4 females, 11 males) with PDD-NOS; both of these diagnoses are included within the ASD category in the DSM-5 (American Psychiatric Association, 2013). As defined in the DSM-IV-TR (American Psychiatric Association, 2000), PDD-NOS includes the heterogeneous subtypes of ASD, ranging from so-called atypical autism to a subgroup with symptoms milder than those of Asperger’s disorder (i.e., satisfying fewer diagnostic criteria than required for a diagnosis of Asperger’s disorder). In this study, only high-functioning PDD-NOS participants with milder symptoms than those associated with Asperger’s disorder were included. The diagnosis was made using the DSM-IV-TR via a stringent procedure in which every item of the ASD diagnostic criteria was investigated in interviews with participants and their parents (and professionals who helped them, if any) by at least two psychiatrists with expertise in developmental disorders. Only participants who met at least one of the four social impairment items (i.e., impairment in nonverbal communication including lack of joint attention, sharing interest, relationship with peers, and emotional and interpersonal mutuality) without satisfying any items of the criteria of autistic disorder, such as language delay, were included. Comprehensive interviews were administered to obtain information on the participant’s developmental histories for diagnostic purposes. Neurological and psychiatric issues other than those associated with ASD were ruled out. Participants were not taking medication. The intelligence quotients (IQs) of all participants in the ASD group had been assessed at other facilities and were reported to be within the normal range. Participants who agreed to newly undergo IQ tests (*n* = 33) were assessed using the revised Wechsler Adult Intelligence Scale, third edition (Nihon Bunka Kagakusha, Tokyo, Japan). All participants in the ASD group were recruited on a voluntary basis at Kyoto University, Rakuwa-kai Otowa Hospital, and the Organization for Promoting Neurodevelopmental Disorder Research. Although two additional individuals planned to participate in the present study, MRI scans of these individuals could not be conducted because macroscopic structural abnormalities due to a previous traffic accident, and artifacts due to orthodontic appliances, respectively, were detected during prescan procedures.

The symptom severity of the participants who were willing to undergo a further detailed interview (*n* = 25) was assessed quantitatively using the Childhood Autism Rating Scale (CARS) (Schopler et al., 1986). The CARS is an effective tool used to evaluate the severity of ASD symptoms in adults, adolescents, and children (Mesibov et al., 1989). The CARS includes 15 items used to assess ASD-related behaviors. Each item is scored from 1.0 to 4.0 in 0.5 increments, with higher scores indicative of more severe symptoms. Total scores range from 15 to 60.

The TD group consisted of 36 adults (11 females, 25 males; mean ± *SD* [range] age = 24.9 ± 5.5 [20–43] years) who were matched for age (*t*-test, *p* > 0.10) and sex (χ^2^-test, *p* > 0.10) with the ASD group. A psychiatrist or psychologist administered a short structured diagnostic interview using the Mini-International Neuropsychiatric Interview (Sheehan et al., 1998); no neuropsychiatric problem was detected in any participant. All participants in the TD group were recruited on a voluntary basis at Kyoto University.

After the procedures were fully explained, all participants provided written informed consent for participation. This study was approved by the local ethics committee of the Primate Research Institute, Kyoto University, and was conducted in accordance with institutional ethical provisions and the Declaration of Helsinki.

### MRI Acquisition

Image scanning was performed using a 3-T MRI system (MAGNETOM Trio, A Tim System, Siemens, Erlangen, Germany) at the ATR Brain Activity Imaging Center using a 12-channel head coil. Small elastic pads were placed on both sides of the head to minimize head motion and on-line visual inspection using a video camera confirmed that none of the participants showed observable head movement. A T1-weighted high-resolution anatomical image was obtained using a magnetization-prepared rapid-acquisition gradient-echo sequence (repetition time = 2,250 ms; echo time = 3.06 ms; inversion time = 1,000; flip angle = 9°; field of view = 256 mm × 256 mm; voxel size = 1 mm × 1 mm × 1 mm).

### Image Analysis

All image analyses were implemented in MATLAB R2012b (MathWorks, Natick, MA, United States). Image preprocessing and VBM analysis were performed using the statistical parametric mapping package, SPM8^1^ and the VBM8 toolbox^2^. First, two of the study authors (WS and TK) independently conducted the visual inspection of T1 images and confirmed no macroscopic lesions or artifacts in the images. Then, image preprocessing was performed using the VBM8 toolbox with default settings. All T1 images were segmented into gray matter, white matter, and cerebrospinal fluid using an adaptive maximum *a posteriori* (AMAP) approach (Rajapakse et al., 1997). Intensity inhomogeneity in the MR image was modeled as slowly varying spatial functions and thus corrected in the AMAP estimation. The segmented images were then used for a partial volume estimation using a simple model with mixed tissue types to improve segmentation (Tohka et al., 2004). Furthermore, a spatially adaptive non-local-means denoising filter was applied to deal with spatially varying noise levels (Manjón et al., 2010). A Markov random field cleanup was used to improve the image quality. The gray matter images in native space were subsequently normalized to the standard stereotactic space defined by the Montreal Neurological Institute using the Diffeomorphic Anatomical Registration with the Exponentiated Lie Algebra algorithm approach (Ashburner, 2007). We used predefined templates provided in the VBM8 toolbox, derived from 550 healthy brains from the IXI-database^3^. The resulting normalized images of the gray matter were modulated using Jacobian determinants with non-linear warping only (i.e., m0 image in the VBM8 outputs) to exclude the effect of total intracranial volume. Finally, the normalized modulated images of the gray matter were resampled to a resolution of 1.5 mm × 1.5 mm × 1.5 mm and smoothed using a 8-mm full-width at half-maximum isotropic Gaussian kernel.

To identify the brain regions associated with differences between the groups, we conducted VBM using a general linear model analysis, with group as the effect-of-interest factor and sex and age as the effect-of-no-interest covariates. The association between group difference and the volume of the gray matter was tested using *T*-statistics and reported as a *Z*-score after the *T*-value was transformed into the standard normal distribution. Voxels were deemed to be statistically significant if they reached the extent threshold of *p* < 0.05, after false-discovery rate (FDR) correction for multiple comparisons based on the topological FDR procedure (Chumbley and Friston, 2009), with a cluster-forming threshold (CFT) of *p* < 0.01 (uncorrected). Note that the main results were unchanged after the non-stationary cluster extent corrections (Hayasaka et al., 2004). According to a recent study (Eklund et al., 2016), the parametric cluster size inference may inflate the false positive rate at a CFT of *p* < 0.01. Hence, to validate our results, we conducted follow-up analyses using Permutation Analysis of Linear Models software^4^ (Winkler et al., 2014), which is a permutation-based inference tool for nonparametric statistics. The threshold for significance was set at *p* < 0.05 after correcting for family wise error using the threshold-free cluster enhancement approach (Smith and Nichols, 2009). Only the effects that reached significance in both of these parametric and nonparametric analyses are reported. We also explored the effects of ASD subtype (Asperger’s disorder vs. PDD-NOS) using these predefined thresholds. The clusters that showed significant group difference (TD > ASD) were used as an inclusive mask.

The brain structures were labeled anatomically according to the Automated Anatomical Labeling atlas (Tzourio-Mazoyer et al., 2002) included in the MRIcron software^5^ and the probabilistic cytoarchitectonic atlas included in the SPM Anatomy toolbox^6^ (Eickhoff et al., 2005). Corresponding Brodmann’s areas were also identified using the MRIcron software. The group difference in gray matter volume was illustrated by plotting the gray matter values extracted at the peak voxels after adjusting for the effects of no interest by regressing out age- and sex-related variance.

To identify the brain network associated with group differences, we performed PLS (McIntosh et al., 1996) using PLSGUI^7^. The normalized images of the gray matter corrected for intracranial volume (i.e., m0 image in the VBM8 outputs) were analyzed. PLS is a multivariate statistical analysis that examines distributed sets of correlated voxels; PLS conducts singular value decomposition for voxels, similar to traditional principal component analyses, but the solutions are constrained to external variables (McIntosh and Lobaugh, 2004). In this study, we searched for the voxel distributions associated with the group differences (ASD vs. TD) using the mean centering PLS analysis. PLS analysis with mean centering is a data-driven version of PLS, in which a set of latent variables (LVs) that explain the maximum covariance between the gray matter volume data and the group effects are derived without explicitly contrasting the two groups. Hence, this type of PLS is referred to as discriminant analysis (Krishnan et al., 2011). The spatial pattern of brain voxels was characterized by voxel saliences indicating the degree to which each voxel was related to each LV (i.e., weight for a given LV).

The statistical significance of the resultant LVs was assessed using permutation tests that were repeated 1,000 times (cf. Krishnan et al., 2011) with a threshold of *p* < 0.01. The reliability of the voxel saliences was determined by estimating their standard error using bootstrap sampling, in which 1,000 random samplings of the participants with replacement allowing duplication were performed (cf. Krishnan et al., 2011). The bootstrap ratio (BSR) was then calculated for each voxel by dividing the voxel salience by its bootstrapped standard error. The threshold of the BSR was set to ±3.3, corresponding approximately to a *p*-value of 0.001.

To evaluate the ability of the PLS analysis to discriminate between the ASD and TD groups, we performed PLS-DA (Barker and Rayens, 2003) with leave-one-out cross-validation using the classification toolbox (Ballabio and Consonni, 2013). The inputs were the gray matter voxels showing significant BSR of the LVs in the above PLS analysis. For a baseline comparison, we also analyzed the whole brain gray matter voxels as inputs. For classification performance, the accuracy ([correctly selected ASD individuals + correctly rejected TD individuals]/[total ASD individuals + total TD individuals]), sensitivity (correctly selected ASD individuals/total ASD individuals), specificity (correctly rejected TD individuals/total TD individuals), and precision (correctly selected ASD individuals/[correctly selected ASD individuals + wrongly selected TD individuals]) were computed.

To explore the association between the regional/network-level gray matter volume reduction and the severity of ASD, we performed correlational analyses. For the overall symptom severity index, we calculated the total CARS score. For the social mulfunctioning index, we also caluculated the sub-total of five items (no. 1, 2, 7, 11, and 12) that were commonly categorized into the social functioning construct in previous studies evaluating the factor structure of the CARS (cf. Magyar and Pandolfi, 2007), as in previous studies (Uono et al., 2011; Yoshimura et al., 2015). To investigate the association between these indices and regional gray matter volume in the ASD group, we conducted VBM analyses using general linear models, with the index (total or social CARS score) as the effect-of-interest factor and sex and age as the effect-of-no-interest covariates. We selected those regions that showed significant group differences in the above VBM analysis as regions of interest. The significance threshold was the same as that in the above VBM analysis. Next, to explore the association between the indices and network-level gray matter volume, we used the brain score derived from the above PLS analysis as the dependent variable and conducted general linear model analyses using the index (total or social CARS score) as the effect-of-interest factor and sex and age as the effect-of-no-interest covariates. The significance threshold was the same as that in the above PLS analysis.

## Results

### Background Information

The demographics and psychiatric information of the ASD and TD groups are summarized in **Table 1**. The total CARS scores in the ASD group were comparable with those of participants with high-functioning ASD, including those with Asperger’s disorder and PDD-NOS, reported in previous studies (*t*-test, *p* > 0.1) (Koyama et al., 2007; Uono et al., 2011; Sato et al., 2012), indicating that symptoms in the ASD group were severe. The full-scale IQs of all participants in the ASD group were within the normal range. All participants were right handed.

**Table 1:** Mean (with *SD* [range]) background information of participants.

Data	ASD						TD	
			
	Total		Asperger		NOS			
*n* Total	36		21		15		36	
Female	11		7		4		11	
Male	25		14		11		25	
Right-handed	36		21		15		36	
Age	27.0	(8.0 [18–53])	27.5	(9.3 [18–53])	26.2	(6.3 [18–42])	24.9	(5.5 [20–43])
IQ^1^ Full-scale	110.4	(13.3 [86–134])	110.2	(12.0 [89–131])	110.7	(15.3 [86–134])		
Verbal	113.3	(14.5 [76–140])	114.8	(13.9 [89–140])	111.2	(15.4 [76–135])		
Performance	104.7	(14.6 [71–128])	102.3	(12.9 [74–121])	107.9	(16.5 [71–128])		
CARS^2^	24.4	(3.6 [17.5–31.5])	25.0	(3.5 [18.5–31.5])	23.5	(3.8 [17.5–29.0])		


*ASD, autism spectrum disorder; TD, typically developing; IQ, intelligence quotient; CARS, Childhood Autism Rating Scale.*

*^1^*n* = 33; ^2^*n* = 25.*

### Voxel-Based Morphometry

**FIGURE 1:**
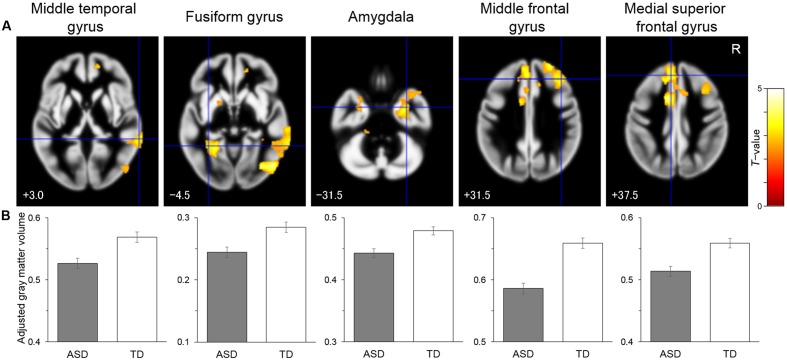
Brain regions showing significantly reduced gray matter volume in the autism spectrum disorder (ASD) group relative to the typically developing (TD) group. **(A)** Statistical parametric maps. The areas are overlaid on the template gray matter image. Numbers below the slices indicate the *z*-axis of the Montreal Neurological Institute system coordinates. The blue crosses indicate the activation foci and the red–yellow color scale represents the *T-*value. R, right hemisphere. **(B)** Mean (with *SE*) adjusted gray matter volume at the peak voxel. Effects of no interest (sex and age) were cavaried out.

To identify the brain regions associated with the differences between the groups, structural MRI data were analyzed by univariate VBM, with group as the effect-of-interest and age and sex as the effect-of-no-interest covariates. The results revealed a significant main effect for group, in which there were reduced volume in the ASD compared with the TD group in the right posterior region including the IOG and MTG, in the left posterior–limbic region including the FG and amygdala, in the right limbic region including the amygdala, in the right lateral prefrontal region covering the IFG, and in the bilateral medial prefrontal region including the right OFC and left DMPFC (**Table 2** and **Figure 1**). There was no significant cluster for a main effect for group indicating a heightened volume for the ASD compared with the TD group. Similarly, an exploration of the effects of ASD subtype did not reveal any significant clusters. A visual inspection of the adjusted gray matter volume (**Supplementary Figure S1**) showed that the values were in the order of Asperger’s disorder <PDD-NOS<TD in some regions (e.g., MTG) and were comparable across the Asperger’s disorder and PDD-NOS groups in other regions (e.g., FG).

**Table 2:** Brain regions that exhibited significantly lower gray matter volume in the autism spectrum disorder (ASD) group than in the typical developing group.

			Coordinates				
						
Area	Region	BA	*x*	*y*	*z*	*Z*-value	Cluster size (mm^3^)	PALM *p*-value	
Lateral occipital–temporal	R. Inferior occipital gyrus	19	50	–75	–3	4.12	15623	0.017	
	R. Inferior temporal gyrus	20	53	–51	–11	3.89		0.022	
	R. Middle temporal gyrus	20	71	–41	–9	3.48		0.024	
	R. Middle temporal gyrus	22	66	–41	3	3.41		0.030	
	R. Middle temporal gyrus	21	69	–29	–3	3.23		0.030	
Ventral temporal–limbic	L. Fusiform gyrus	37	–33	–50	–5	3.19	10702	0.058	+
	L. Amygdala	–	–18	3	–14	3.21		0.080	+
Limbic	R. Amygdala	–	29	–12	–32	3.50	10800	0.045	
	R. Parahippocampal gyrus	20	33	–11	–29	3.20		0.048	
Lateral prefrontal	R. Middle frontal gyrus	9	36	27	53	3.31	7830	0.043	
	R. Middle frontal gyrus	46	27	53	32	3.70		0.036	
Medial prefrontal	R. Supplementary motor area	6	11	6	48	3.79	21769	0.025	
	L. Middle cingulate gyrus	32	–11	9	42	4.50		0.017	
	R. Middle cingulate gyrus	32	12	18	41	3.78		0.034	
	L. Medial superior frontal gyrus	8	–3	33	50	3.77		0.011	
	L. Anterior cingulate gyrus	32	–12	33	29	2.89		0.035	
	L. Middle frontal gyrus	9	–23	35	44	2.64		0.040	
	L. Medial superior frontal gyrus	32	–8	41	38	3.92		0.010	
	R. Medial orbital gyrus	11	17	50	–2	3.15		0.053	+


*R, Right; L, Left; BA, Brodmann’s area; PALM, Permutation Analysis of Linear Models. ^+^Marginally significant (significant voxels were detected in adjacent regions).*

### PLS and PLS-DA

**FIGURE 2:**
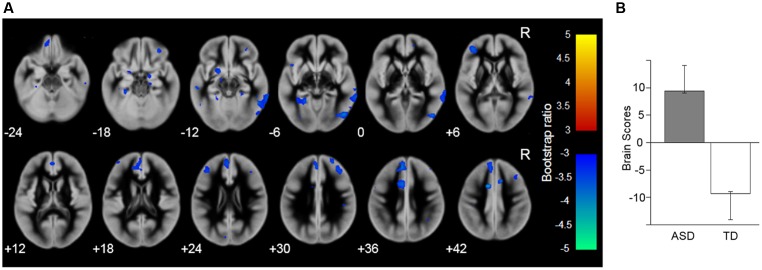
Brain network showing the latent variable that significantly discriminated the autism spectrum disorder (ASD) and typically developing (TD) groups. **(A)** Latent variable maps. The blue–cyan color indicates the reduced gray matter volume in the ASD compared with the TD group; the red–yellow color indicates the opposite direction. The areas are overlaid on the template gray matter image. Numbers below the slices indicate the *z*-axis of the Montreal Neurological Institute system coordinates. R, right hemisphere. **(B)** The mean (with the upper and lower 95% confidence limits) brain scores of the latent variable (i.e., the degree to which the pattern of gray matter volume across the whole brain, identified by the latent variable, is expressed in each participant).

To identify the brain network (i.e., the sets of correlated voxels) associated with the group differences, PLS analysis was performed for the gray matter images. The results revealed that the first LV showed significant discrimination between the groups (*p* = 0.003; **Figure 2**) and explained 100% of the crossblock covariance. The spatial distribution of brain voxels that negatively weighted on this LV (i.e., ASD < TD) included all of the social brain regions that showed group differences in the above regional gray matter volume analyses (i.e., the right IOG, left FG, right MTG, bilateral amygdala, right IFG, right OFC, and left DMPFC).

**FIGURE 3:**
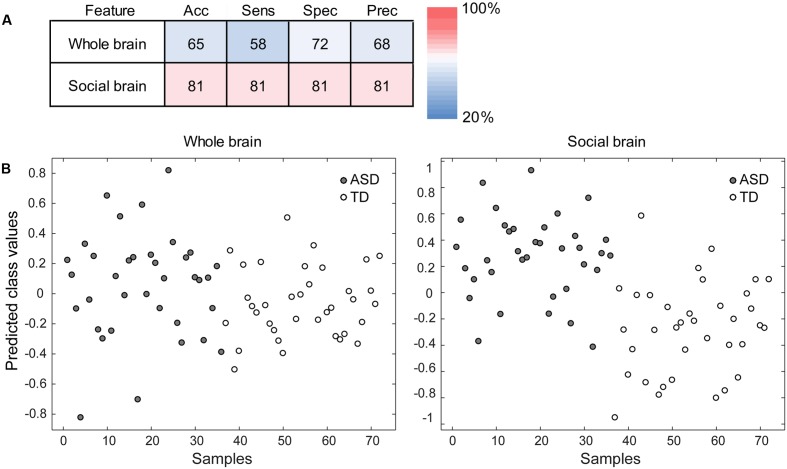
Performance of the partial least squares (PLS) discriminant analysis (PLS-DA). **(A)** The accuracy (Acc), sensitivity (Sens), specificity (Spec), and precision (Prec) of the analysis using the whole brain voxels (the baseline condition; upper) and those of the analysis using the gray matter voxels in the PLS latent variable (i.e., the social brain network; lower) are shown. The red–blue color indicates the percentage. **(B)** The discrimination plots for each participant in the autism spectrum disorder (ASD) and typically developing (TD) groups in the PLS-DA using the whole brain voxels (left) and social brain network voxels (right). The *x*-axis represents individual participants, and the *y*-axis represents the predicted class values that were estimated during the cross-validation. The more positive (or negative) the value on the *y*-axis, the better the discrimination performance for the ASD (or TD) group.

To evaluate the ability of the PLS analysis to discriminate between the ASD and TD groups, PLS-DA was performed using information from the LV derived from the PLS analysis. The analysis using the LV information showed sufficiently high accuracy, sensitivity, specificity, and precision (all >80%) compared with the analysis using the whole brain voxels as input information (**Figure 3**).

### Relationship between Regional/Network-Level Gray Matter Volume and Symptom Severity

To explore the relationship between ASD symptom severity and the reduction in the regional/network-level gray matter volume identified by the above VBM/PLS analyses, we performed general linear model analyses using the total CARS score or social CARS score as the effect-of-interest independent variable. The effects of age and sex were covariated out. However, VBM analysis did not detect any significant association between the total/social CARS score and regional gray matter volume in the ASD group. The general linear model using the PLS brain score as the dependent variable also did not show a significant association between the total/social CARS score and the reduction in network-level gray matter.

## Discussion

Our univariate VBM analysis revealed that gray matter volume in several brain regions was reduced in the ASD adult group compared with the TD adult group. These regions included the IOG, FG, MTG, amygdala, IFG, OFC, and DMPFC. Since these regions are considered to be the social brain regions (Brothers et al., 1990; Adolphs, 2003; Blakemore, 2008), our results are consistent with the theoretical proposal that social brain regions are impaired in ASD (Emery and Perrett, 2000; Johnson et al., 2005; Frith, 2007; Pelphrey and Carter, 2008). The results are also in line with those of several previous functional neuroimaging studies reporting reduced activities in these regions during the processing of social stimuli among individuals with ASD. For example, Sato et al. (2012) reported reduced activity in the IOG, FG, MTG, amygdala, IFG, and DMPFC among ASD compared with TD participants during the observation of dynamic facial expressions. Our results are also consistent with the findings of several previous structural MRI studies that tested adults with ASD and reported reduced gray matter volume in these regions (Abell et al., 1999; Hadjikhani et al., 2006; Craig et al., 2007; Toal et al., 2010; Scheel et al., 2011; Ecker et al., 2012; Mueller et al., 2013). For example, Abell et al. (1999) reported reduced gray matter volume in an ASD compared with a TD group in the DMPFC. However, only a few studies have reported problems in multiple social brain regions, and few of these have applied a correction for multiple comparisons (Duerden et al., 2012; Yang et al., 2016). In the current study, we used a correction for multiple comparisons, thereby providing robust evidence. Although several previous studies have reported null findings, the discrepancy between those studies and the present results may be explained by differences in the ASD group sampled. We evaluated only individuals who were diagnosed with Asperger’s disorder or a milder type of PDD-NOS. Therefore, we controlled for the effect of language impairments, which could strongly affect gray matter structures in widespread brain regions (Lai et al., 2015; Sharda et al., 2017). In addition, we controlled for other confounding factors, such as comorbidities and medications, which have not been controlled for or reported in some previous studies. Furthermore, the size of our ASD group was relatively large compared with those of several previous studies. Although the present exploratory analysis did not find a significant effect of ASD subgroup, the profiles of the adjusted gray matter volume in some social brain regions showed lower values in the Asperger’s disorder group than in the PDD-NOS group. This result is consistent with the clinical conditions, such as the PDD-NOS group exhibiting milder ASD symptoms than the Asperger’s disorder group, and suggests the involvement of these regions in ASD symptoms. Taken together, the present study accounted for several potential confounding factors, including language impairments, and showed that a decrease in gray matter volume in social brain regions is partly responsible for ASD.

Furthermore, our multivariate PLS analysis revealed that the network (i.e., the sets of correlated voxels) showed decreased gray matter volume in the ASD relative to the TD group. The network included all the brain regions related to the processing of social stimuli, suggesting that the network represents the social brain network. The correspondence between the results of the VBM and PLS analyses does not always occur because voxels can show an overall decrease without being correlated across individuals. The PLS-DA confirmed that our approach clearly discriminated between the ASD and TD groups. These results are consistent with the notion that the network of the social brain regions is impaired in ASD (Emery and Perrett, 2000; Johnson et al., 2005; Frith, 2007; Pelphrey and Carter, 2008). These results are also consistent with those of several functional neuroimaging studies that have reported reduced functional connectivity in individuals with ASD while engaging in some social tasks, such as facial expression processing (Welchew et al., 2005; Wicker et al., 2008; Sato et al., 2012), face perception (Kleinhans et al., 2008; Koshino et al., 2008; Hoffmann et al., 2016; Lynn et al., 2016), and mindreading (Castelli et al., 2002). However, structural neuroimaging evidence has been lacking. To our knowledge, this is the first reported structural neuroimaging evidence showing reduced gray matter volume in the social brain network in adults with ASD.

Our result showing that the gray matter volume in the social brain network is reduced in adults with ASD has theoretical and practical implications. Theoretically, the network includes widespread brain regions, which are assumed to serve to different functions, such as perception (the IOG, FG, and MTG; Haxby et al., 2000), emotion (the amygdala and OFC; Calder et al., 2001; Rolls, 2004), motor (the IFG; Rizzolatti et al., 2001), and cognition (the DMPFC; Frith and Frith, 2003). Regarding the behavioral and/or neural mechanisms underlying social malfunctioning in ASD, several theories have been proposed. For example, at a behavioral level, whereas some researchers have pointed out the importance of emotional deficits in individuals with ASD, such as empathic responding (Hobson, 2002), other researchers have proposed impairment in cognitive processing, such as mindreading (Baron-Cohen et al., 1985). At a neural level, researchers proposed that the structural and/or functional abnormalities may be found in the perceptual (Grelotti et al., 2002), emotional (Bachevalier, 1994), motor (Williams et al., 2001), or cognitive (Frith, 2001) processing-related brain regions in individuals with ASD. The present findings suggest that these divergent social processes are related and are collectively impaired in ASD. Given that social stimuli are complex in nature, it is possible that processing them requires coordination of multiple different computations, which may be holistically difficult in individuals with ASD.

Practically, our results suggest that structural MRI can provide neuroimaging-based biomarkers for ASD. It has been pointed out that such biomarkers could be clinically useful in complementing or improving the behavioral diagnosis of ASD (Walsh et al., 2011). Additionally, the idea of social brain network impairment in ASD suggests that behavioral social malfunctioning in individuals with ASD may be modified by neural or behavioral treatments targeting these social brain regions. Consistent with this, previous studies have reported that electrical stimulation of the amygdala (Sturm et al., 2013) and magnetic stimulation of the OFC (Enticott et al., 2014) modified the behavioral social problems in individuals with ASD. Behavioral training in facial expression communication in individuals with ASD reportedly improved their expression recognition performance and increased neural activity in several social brain regions, such as the MTG and amygdala, during their observation of facial expressions (Bölte et al., 2015). Training in facial communication in TD individuals also increased the gray matter volume in the FG (Kreifelts et al., 2013). Promising directions for further investigation include such practical applications based on the impaired social brain network theory of ASD.

Several limitations to this study should be acknowledged. First, inconsistent with our prediction, an association between the total/social CARS score and decrease in the regional/network-level gray matter volume in the ASD group was not observed. This null finding may be explained by the items included in the CARS. Although the CARS is reliable and valid and one of the most commonly used scales used to evaluate the degree and profiles of ASD (Koyama et al., 2007), it was not developed specifically to quantify the severity of social malfunctioning in individuals with ASD. The use of different assessment tools more specific to social functioning in ASD, such as the social responsiveness scale (Constantino et al., 2003), may be more appropriate for future studies.

Second, because we tested only adult participants, the developmental trajectory of the reduced gray matter volume in the social brain network in ASD remains unknown. Some structural MRI studies in children with ASD have reported the atypical development of global and/or local brain volume (for a review, see Sacco et al., 2015). Based on these data, some researchers proposed theoretical models regarding the atypical development of the social brain network in ASD (e.g., Chevallier et al., 2012; Elsabbagh and Johnson, 2016), but there has been little empirical investigation. Future studies are necessary to investigate the structural development of the social brain network in ASD.

In summary, our VBM analysis showed that the regional gray matter volume was lower in several brain regions in the ASD compared with the TD group, including the IOG, FG, MTG, amygdala, IFG, OFC, and DMPFC. Furthermore, our PLS analysis and PLS-DA revealed a widespread network including these regions discriminated the groups. These results suggest that reduced gray matter volume in the social brain network represents the neural underpinnings of behavioral social malfunctioning in adults with ASD.

## Author Contributions

WS, TK, SU, and MT designed the research; WS, TK, SU, SY, YK, RS, MS, and MT obtained the data; WS, TK, SU, SY, and MT analyzed the data; and all authors wrote the manuscript. All authors read and approved the final manuscript.

## Conflict of Interest Statement

The authors declare that the research was conducted in the absence of any commercial or financial relationships that could be construed as a potential conflict of interest.

## Footnotes

1 http://www.fil.ion.ucl.ac.uk/spm

2 http://dbm.neuro.uni-jena.de

3 http://www.brain-development.org

4 https://fsl.fmrib.ox.ac.uk/fsl/fslwiki/PALM

5 http://www.mccauslandcenter.sc.edu/mricro/mricron/

6 http://www.fz-juelich.de/inm/inm-1/DE/Forschung/_docs/SPMAnatomyToolbox/SPMAnatomyToolbox_node.html

7 http://www.rotman-baycrest.on.ca/pls/

## Supplementary Material

**FIGURE S1 |** Mean (with *SE*) adjusted gray matter volume in the Asperger’s disorder (ASP), pervasive developmental disorder not otherwise specified (NOS), and typically developing (TD) groups. Values of the peak voxels showing significantly reduced gray matter volume in the autism spectrum disorder group compared with the TD group are shown. The effects of no interest (sex and age) were cavaried out.
