# Supplementary figures and images for: Reduced Gray Matter Volume in the Social Brain Network in Adults with Autism Spectrum Disorder
Source: Front Hum Neurosci. 2017 Aug 4;11:395. doi: 10.3389/fnhum.2017.00395 (PMC5543091; doi:10.3389/fnhum.2017.00395)

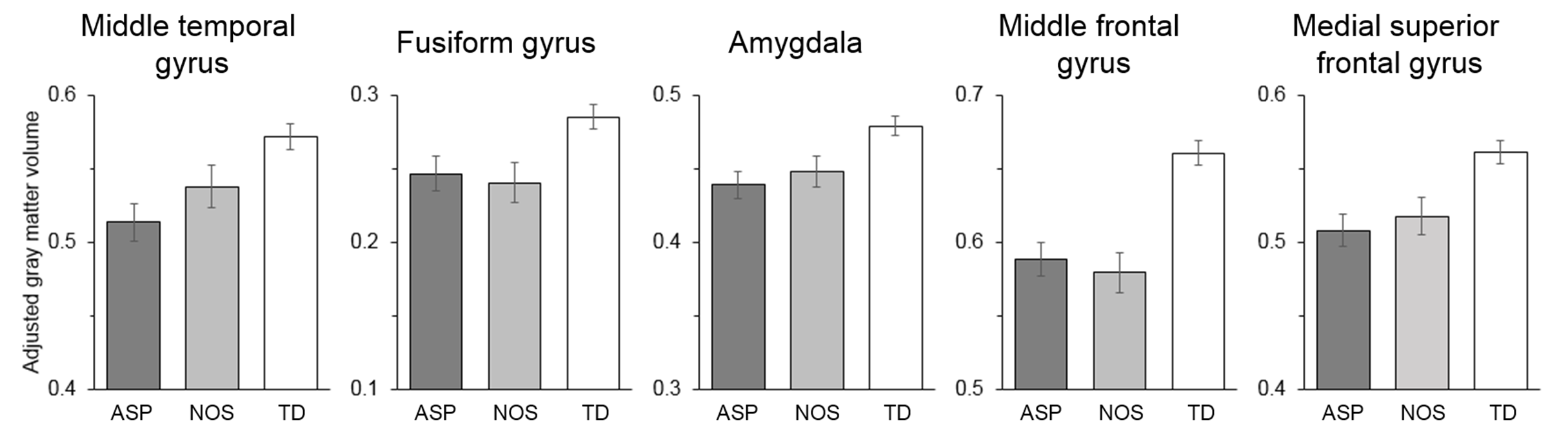

Supplement: Supplementary file 1 [file Image_1.TIF]
